# Evaluating the Psychometric Properties of the 7C Vaccination Readiness Scale: Evidence from Slovenia

**DOI:** 10.2478/sjph-2026-0004

**Published:** 2026-03-01

**Authors:** Mitja Vrdelja, Stefani Branilović, Monika Lamot, Andrej Kirbiš

**Affiliations:** National Institute of Public Health, Trubarjeva cesta 2, 1000 Ljubljana, Slovenia; University of Maribor, Faculty of Arts, Department of Sociology, Koroška cesta 160, 2000 Maribor, Slovenia

**Keywords:** Vaccine hesitancy, 7C, Vaccination readiness, Psychometric properties, Slovenia, oklevanje glede cepljenja lestvica, 7C, pripravljenost na cepljenje, psihometrične lastnosti, Slovenija

## Abstract

**Introduction:**

Vaccine hesitancy remains a major global public health challenge. Psychological models, such as the 7C vaccination readiness scale, aim to identify key psychological determinants of vaccine uptake. While the scale has shown validity in various cultural contexts, its psychometric properties have not yet been evaluated in Slovenia.

**Methods:**

This study assessed the psychometric properties, convergent validity, and criterion validity of the Slovenian version of the 7C scale using a representative sample of 1,350 adults via confirmatory factor analysis (CFA), correlation coefficients, and regression analyses.

**Results:**

The bifactor model showed mixed psychometric properties. CFA revealed a weak model fit, with two items showing inadmissible estimates; these were removed. The revised model showed improved estimation and acceptable, though still suboptimal, fit indices. Convergent validity was supported by significant correlations between the general vaccination readiness factor and conspiracy beliefs, while individual components showed weaker associations. Criterion validity analyses showed that the general factor was the strongest predictor of vaccination intention, with calculation and compliance also contributing. The 7C model explained more variance in vaccination intention than the 5C model, suggesting greater utility. Despite structural limitations, the scale demonstrates practical value and offers recommendations for refinement.

**Conclusions:**

The Slovenian version of the 7C scale proved to be a valuable tool for predicting vaccination intention. The general factor was a robust predictor, and calculation and compliance showed additional validity. However, components like complacency and constraints need revision to improve model fit. With refinement, the 7C scale holds promise for research and public health applications across contexts.

## Izvleček

**Uvod:**

Oklevanje glede cepljenja ostaja pomemben globalni izziv za javno zdravje. Psihološki modeli, kot je lestvica pripravljenosti na cepljenje 7C, poskušajo identificirati ključne psihološke dejavnike, ki prispevajo k precepljenosti. Čeprav je bila veljavnost lestvice 7C potrjena v različnih kulturnih kontekstih, njene psihometrične značilnosti v Sloveniji še niso bile preučene.

**Metode:**

V raziskavi smo preučili psihometrične lastnosti ter konvergentno in kriterijsko veljavnost slovenske različice lestvice 7C na reprezentativnem vzorcu 1350 odraslih s pomočjo potrditvene faktorske analize (CFA), korelacijskih koeficientov in hierarhične regresijske analize.

**Rezultati:**

Bifaktorski model lestvice pripravljenosti na cepljenje je pokazal mešane psihometrične značilnosti. Potrditvena faktorska analiza je pokazala nekoliko šibko prileganje modela, pri čemer sta dva elementa pokazala nedopustne ocene, zato sta bila odstranjena. Revidirani model je pokazal izboljšano oceno in sprejemljive, čeprav še vedno neoptimalne indekse prileganja. Konvergentna veljavnost je bila potrjena s statistično značilnimi korelacijami med splošnim faktorjem pripravljenosti na cepljenje in prepričanji o teorijah zarot, medtem ko so posamezne komponente 7C kazale manjše povezave s prepričanji o zarotah. Analize kriterijske veljavnosti so pokazale, da je splošni faktor najmočnejši napovednik statusa cepljenja, značilno pa sta k napovedovanju prispevala tudi preračunljivost in skladnost. Model 7C je pojasnil večji delež variance kot model 5C, kar nakazuje na večjo napovedno uporabnost. Kljub strukturnim omejitvam lestvica 7C kaže praktično vrednost, ob tem pa se priporoča teoretična nadgradnja v prihodnjih raziskavah.

**Zaključki:**

Kljub nekaterim strukturnim omejitvam se je slovenska različica lestvice 7C pripravljenosti na cepljenje izkazala za uporabno orodje za napovedovanje cepilnega statusa. Splošni faktor se je izkazal za robusten in konsistenten napovednik, medtem ko sta komponenti preračunljivost in skladnost prav tako izkazali konvergentno in kriterijsko veljavnost. Ugotovitve kažejo na potrebo po reviziji nekaterih komponent, zlasti samozadostnosti in omejitev, z namenom izboljšanja teoretične skladnosti in prileganja modela. Z nadaljnjim izpopolnjevanjem ima lestvica 7C velik potencial tako za raziskovalne kot javnozdravstvene namene v različnih kulturnih kontekstih.

## 1 INTRODUCTION

Vaccine uptake remains one of the most crucial tools for managing public health threats and preventing the spread of infectious diseases (1, 2). However, widespread vaccine hesitancy, which is defined as the delayed acceptance or refusal of vaccines despite their availability, has become a major global concern (3,4,5), including in Slovenia (6, 7). While access to vaccines is essential, actual vaccination rates are also heavily influenced by psychological and social factors. Vaccine readiness “includes components that increase or decrease the likelihood of getting vaccinated” (8) and plays a key mediating role in vaccine uptake (8). Several questionnaires have been developed to assess psychological aspects of vaccination-related attitudes and readiness. Widely used instruments include the Parent Attitudes about Childhood Vaccines (PACV) scale (9,10,11), the Vaccination Confidence Scale (12), and the Vaccination Attitudes Examination (VAX) scale (13,14,15). These measures primarily assess specific attitudinal facets, such as confidence in vaccines, safety concerns, or distrust in vaccine-related institutions, but do not conceptualise vaccination readiness as a broad, component-based construct. In recent years, psychological scales such as the 5C (16, 17) and 7C (8, 18, 19), where “C” stands for “component”, have been developed to capture key antecedents of vaccination behaviour. These questionnaires allow researchers and policymakers to understand better not only why people may refuse vaccines, but also how to encourage uptake through targeted interventions. To date, none of these multidimensional instruments has been validated in Slovenian. To address this gap, the present study evaluates the 7C scale, which operationalises vaccination readiness using a comprehensive component-based approach.

The 5C model conceptualises vaccination decisions in terms of five psychological components: confidence, complacency, constraints, calculation, and collective responsibility (16). The 7C scale represents an extension of the earlier 5C model by incorporating additional psychological components, namely compliance and conspiracy endorsement, thereby offering improved explanatory and predictive power (8, 18). The 7C vaccination readiness model, therefore, consists of seven components that reflect psychological drivers and barriers to vaccine-related behaviour: confidence, complacency, constraints, calculation, collective responsibility, compliance, and conspiracy (8, 20). Confidence refers to trust in vaccine effectiveness and safety, as well as in health authorities. Complacency reflects the belief that vaccination is unnecessary because of a low perceived threat from diseases. Constraints encompass both structural and psychological barriers to vaccination, such as cost, access, and time constraints. Calculation involves weighing risks and benefits before making a decision, while collective responsibility denotes the willingness to protect others. Compliance refers to adherence to social norms or regulations regarding vaccination, and conspiracy refers to belief in misinformation or distrust in authorities (8, 20, 21). Together, these components capture the complex psychological terrain that shapes vaccine decisions.

Several empirical studies have confirmed the reliability and validity of the 7C scale in diverse cultural contexts (e.g. Germany, Japan, France, Croatia, Denmark). For example, Geiger et al. (8) showed that both the original 21-item scale and its 7-item short form achieved excellent factorial fit, providing strong evidence of structural validity. Similarly, Tokiya et al. (22) validated the Japanese version of the scale among parents, finding that lower 7C scores were associated with lower vaccination rates among children. Machida, Kojima et al. (4) also confirmed acceptable model fit using confirmatory factor analysis, although the calculation subscale showed lower reliability. Oudin Doglioni et al. (18) further demonstrated the predictive utility and superior model fit of the 7C compared to the 5C scale across multiple population groups in France. These findings underscore the 7C scale's utility for capturing the psychological determinants of vaccine behaviour, though further testing is needed in underrepresented national contexts, such as Slovenia. At the same time, previous validation studies in other languages, including German and Japanese versions, have raised concerns about the scale's psychometric properties, particularly the reliability and stability of certain components (4, 23). These limitations are explicitly acknowledged in the present study.

Furthermore, demographic and socioeconomic factors have been identified as significant predictors of the 7C vaccination readiness scale, although they remain relatively underexplored. Hansen et al. (19), using a representative Norwegian adult sample (N = 4,137, 35 years old and older), found that lower 7C scores were associated with being male, younger age, lower education, and lower income. Similarly, Kalebić Maglica and Šincek (24), in a study among Croatian individuals (N = 1,769, aged 18 to 77 years), reported that those with higher education, higher income, older age, and female gender exhibited higher overall 7C readiness. Santana et al. (21), in a Danish study of parents (N = 2,941; Mage = 35.2, SD = 5.7), found that older parents expressed lower confidence and collective responsibility, perceived more constraints, and held stronger conspiracy beliefs. Moreover, male parents showed higher complacency, while those with higher education levels reported lower complacency, fewer perceived constraints, and lower agreement with conspiracy beliefs. These findings suggest that demographic patterns must be taken into account when validating the 7C scale.

The present study aimed to examine the psychometric properties (e.g., 18, 19, 25, 26) of the Slovenian version of the 7C vaccination readiness scale. Specifically, building on the scale's strong theoretical foundation and demonstrated predictive validity across diverse cultural settings, the study assessed its internal consistency, construct validity, and convergent and criterion validity in a representative adult sample from Slovenia. Accordingly, the present study addresses the following guiding research question (RQ): RQ: Does the Slovenian version of the 7C vaccination readiness scale demonstrate appropriate factor structure, criterion and convergent validity?

## 2 METHODS

### 2.1 Sample

The study was conducted on a representative sample of adult residents of Slovenia. A total of 1,350 participants completed the first wave of an online survey in June 2025. The sample was stratified by gender, age group, education level, and region to ensure national representativeness. The study received ethical approval from the Commission for Deontological and Ethical Issues of the National Institute of Public Health (No. 631-2/2025-18 (013)). The questionnaire was administered between June 3 and June 12, 2025, via Valicon's web-based survey platform (AI-Assisted Interviewing system). Participants were recruited through a probability-based online panel. Invitations to participate were distributed via email, containing a personalised survey link. In total, 3,422 individuals were invited to participate in the study. Of these, 1,622 invitees clicked the survey link, and 1,350 respondents completed the questionnaire, yielding an overall response rate of 39%. A total of 104 invited individuals were unable to participate due to quota fulfilment. All completed responses were retained for analysis; no partially completed questionnaires were included in the final sample. All collected data were anonymous, with access limited to researchers at the National Institute of Public Health (NIJZ), the data collection agency Valicon, and collaborating academics. The study was not pre-registered.

Participants' ages ranged from 18 to 74 years, with a mean age of 47.48 years (SD=15.44). The sample included 51.5% male and 48.5% female participants. In terms of education, the largest group had completed secondary professional education (35.2%), followed by general secondary education (15.4%) and lower vocational education (15%). Regarding economic self-assessment, 38.8% of respondents reported that paying for essential expenses was neither easy nor difficult, while 27% reported they could do so quite easily. A detailed overview of the sample's demographics is presented in Table 1.

**Table 1.** Sample characteristics.

		**f**	**%**
**Gender**	Male	696	51.5
	Female	654	48.5

**Education**	Incomplete primary education	3	0.2
	Primary education	51	3.8
	Lower or secondary vocational education	203	15.0
	Secondary professional education	476	35.2
	General secondary education	208	15.4
	Higher vocational education, post-secondary education (previous higher education, 2 years + diploma)	83	6.2
	Higher vocational education, post-secondary education (former VS - 3 years, 1st Bologna degree)	107	7.9
	University degree (4-can be 6 years + diploma)	123	9.1
	Bologna Master's degree	42	3.1
	Specialisation	2	0.2
	Master's degree	25	1.9
	Doctorate	19	1.4

**Economic situation**	Very difficult	53	3.9
	Quite difficult	175	13.0
	Neither difficult nor easy	523	38.8
	Quite easy	365	27.0
	Very easy	220	16.3

**Age**		M	SD
		47.48	15.44

Note: Economic situation was assessed using the following question: “Considering the total income of your household (including all income sources of all household members), how difficult or easy was it for your household to cover all essential expenses during the past two months?” Responses were recorded on a 5-point scale ranging from very difficult to very easy, with higher values indicating a more favourable perceived economic situation.

### 2.2 Measures

#### 2.2.1 7C

The Slovenian version of the 7C vaccination readiness scale was developed by the National Institute of Public Health of Slovenia (NIPH) following a standardised forward-backwards translation procedure. First, two independent bilingual translators translated the original English version into Slovenian. The two forward translations were then synthesised into a single version through discussion. This synthesised version was subsequently back-translated into English by two independent translators who were blind to the original instrument. The back-translation was compared with the original English version to identify any discrepancies in meaning. A committee reviewed all versions to reach consensus on the final Slovenian version. The questionnaire was pilot-tested with a small sample to assess clarity and comprehension prior to administration in the main study.

Each of the seven components of the 7C scale was measured with three items, yielding a total of 21 items. All items were rated on a 7-point Likert scale from 1 (completely disagree) to 7 (completely agree). The items for each of the 7C components are presented in Table 2. Items marked with (R) were reverse-scored so that higher values consistently indicate higher vaccination readiness. In the original validation study, the 7C scale demonstrated good overall reliability, with a McDonald's omega of 0.88 for the general vaccination readiness factor. Reliability estimates for the specific components ranged from ω = 0.27 to ω = 0.59 (8). In the present study, model-based composite reliability, estimated from a bifactor confirmatory factor analysis, indicated good reliability for the general vaccination readiness component (CR = 0.84). Composite reliability estimates for the specific components ranged from CR = 0.18 (Constraints), CR = 0.40 (Complacency), CR = 0.19 (Collective responsibility), CR = 0.33 (Compliance), CR = 0.50 (Conspiracy), to CR = 0.71 (Calculation), consistent with the original validation study.

**Table 2.** Measuring items for 7C vaccination readiness scale.

**Components**	**Item**
**Confidence**	1. Vaccination side effects occur rarely and are not severe for me.
	2. Political decisions about vaccinations are scientifically grounded.
	3. I am convinced that appropriate authorities do only allow effective and safe vaccines.
**Complacency**	4. I do not need vaccinations because infectious diseases do not hit me hard. (R)
	5. Vaccination is unnecessary for me because I rarely get ill anyway. (R)
	6. I get vaccinated because it is too risky to get infected.
**Constraints**	7. I make sure to receive the most important vaccinations in good time.
	8. Vaccinations are so important to me that I prioritise getting vaccinated over other things.
	9. I sometimes miss out on vaccinations because vaccinations are bothersome. (R)
**Calculation**	10. I get vaccinated when I do not see any disadvantages for me. (R)
	11. I only get vaccinated when the benefits clearly outweigh the risks. (R)
	12. For each vaccine, I carefully consider whether I need it. (R)
**Collective responsibility**	13. I also get vaccinated because protecting vulnerable risk groups is important to me.
	14. I see vaccination as a collective task against the spread of diseases.
	15. I also get vaccinated because I am thereby protecting other people.
**Compliance**	16. It should be possible to exclude people from public activities (e.g., concerts) when they are not vaccinated against a specific disease.
	17. The health authorities should use all possible means to achieve high vaccination rates.
	18. It should be possible to sanction people who do not follow the vaccination recommendations by health authorities.
**Conspiracy**	19. Vaccinations cause diseases and allergies that are more serious than the diseases they are supposed to protect from. (R)
	20. Health authorities knuckle under to the power and influence of pharmaceutical companies. (R)
	21. Vaccinations contain chemicals in toxic doses. (R)

Note: Geiger et al. (8).

#### 2.2.2 Vaccination intention

To assess individuals' intention to get vaccinated, we used a question: “How likely is it that in the future you will get vaccinated against COVID-19 on a seasonal basis (in the same way that we can be vaccinated against the flu on a seasonal basis)?”, which was measured on a 5-point scale (1 = not at all likely; 5 = very likely) (similarly in 27, 28).

#### 2.2.3 Conspiracy beliefs

Belief in conspiracy theories was assessed through the question: “Certain political and social events are often the subject of debate (e.g., the attack on the USA on 11 September 2001, the death of Princess Diana, the assassination of John F. Kennedy, etc.). There are suggestions that the ‘official version’ of these events may be an attempt to hide the truth from the public. This ‘official version’ could conceal that these events were planned and secretly prepared by a hidden alliance of powerful individuals or organisations (e.g., secret services or governments). What do you think?”, measured on a 9-point scale (1 = not true at all; 9 = absolutely true).

### 2.3 Statistical analyses

In the statistical analyses, we followed the approach of Geiger et al. (8). To assess the validity and reliability of the 7C vaccination readiness scale, we first evaluated construct validity using a bifactor confirmatory factor analysis (CFA). In this model, all 21 items load on a general component representing overall vaccination readiness, while subsets of items simultaneously load on six orthogonal specific components corresponding to the 7C components other than confidence (i.e., complacency, constraints, calculation, collective responsibility, compliance, and conspiracy). The general component captures variance common to all items, whereas the specific components represent residual variance associated with each component beyond the general readiness dimension. This modelling approach reflects the theoretical structure of the 7C scale, which conceptualises vaccination readiness as a broad latent construct that manifests through multiple, more specific psychological components. The bifactor specification allows for a direct examination of whether a strong general vaccination readiness factor underlies responses to all items, while still accounting for component-specific variance. Model fit was evaluated using common model fit indices, following Hu and Bentler (29): chi-square (χ^2^), Comparative Fit Index (CFI > 0.90), Tucker-Lewis Index (TLI > 0.90), Root Mean Square Error of Approximation (RMSEA < 0.08), and Standardised Root Mean Square Residual (SRMR < 0.08). Convergent validity was evaluated by correlating the 7C latent components with conspiracy beliefs. Finally, criterion validity was assessed by comparing the proportion of variance explained (R^2^) in vaccination intention using both the original 5C and revised 7C models. Specifically, vaccination intention was regressed on the respective set of psychological components in separate models, and the explained variance was compared to evaluate whether the inclusion of the additional components in the 7C framework provided incremental predictive validity over the 5C model, following the analytic strategy proposed by Geiger et al. (8). Therefore, the 5C framework was used as a conceptual benchmark for the development and evaluation of the 7C scale, rather than as a separate object of evaluation. In our analyses, we used the statistical software R (version 2024.12.0+467). All models were estimated using the robust maximum likelihood estimator (MLR) to account for potential deviations from multivariate normality. Missing data were handled using full information maximum likelihood (FIML), which allows for the inclusion of all available data under the assumption that data are missing at random.

## 3 RESULTS

### 3.1 Construct validity

Construct validity was assessed via confirmatory factor analysis (CFA), in which we tested whether the 7-component bifactor structure provided good model fit. The results showed weak model fit (χ^2^ = 20377.414, p < 0.01, CFI = 0.905, TLI = 0.884, RMSEA = 0.091, SRMR = 0.074), as the model fit indices were below the suggested thresholds. During the model evaluation, two items were identified as problematic. The item “Vaccination is unnecessary for me because I rarely get ill anyway.” (assigned to the complacency component) showed a standardised factor loading on the specific factor exceeding 1.0 and a negative residual variance (−2.04), indicating statistical inadmissibility and likely model misfit. The second item, “I sometimes miss out on vaccinations because vaccination is bothersome.” (assigned to the constraints component), demonstrated an almost zero and slightly negative residual variance (−0.04), coupled with an unusually high standardised loading on the specific factor (0.943), suggesting over-saturation and potential redundancy with the general component. To ensure model stability and interpretability, both items were removed from subsequent analyses. We therefore conducted an additional CFA to assess model fit without these two items. After the exclusion of the two items, the revised bifactor model showed no further estimation issues, with all standardised loadings below 1 and all residual variances positive. The results of the revised bifactor model fit (CFI=0.904, TLI=0.880, RMSEA=0.075, SRMR=0.065) showed somewhat weak, but still acceptable model fit indices for the bifactor model (30, 31).

### 3.2 Convergent validity

The results for convergent validity, tested by correlating the 7C scale's modified bifactor structure with the conspiratorial thinking variable, are presented in Table 3. As expected, conspiratorial thinking showed a strong negative correlation with the general component (r = −0.364, p < 0.01), indicating that individuals with higher general vaccination readiness were less likely to endorse conspiratorial beliefs. The 7C conspiracy factor also showed a significant negative correlation with conspiratorial thinking (r = −0.240, p < 0.01), supporting its convergent validity. The calculation component was weakly but significantly negatively correlated (r = −0.076, p < 0.05). On the other hand, the component of collective responsibility showed a modest positive association (r = 0.190, p < 0.01). Overall, these findings provide solid support for convergent validity (RQ).

**Table 3.** Convergent validity of the 7C vaccination readiness scale.

	**Conspiratorial thinking**
**G**	−0.364^**^
**Complacency**	1.602
**Constraints**	1.486
**Calculation**	−0.076^*^
**Collective responsibility**	0.190^**^
**Compliance**	0.026
**Conspiracy**	−0.240^**^

Legend:

* p < 0.05,

** p < 0.01, G = general vaccination readiness component. Correlation coefficients for Complacency and Constraints exceed the typical −1 to +1 range, indicating instability in these specific factors within the bifactor model structure.

### 3.3 Criterion validity

The results for criterion validity, which was assessed using regression analyses examining the impact of the bifactor 7C and 5C scales on vaccination intention, are presented in Table 4. The results suggest that the general component is a strong and highly significant predictor of vaccination intention in both models (β = 0.662, p < 0.01 for 7C; β = 0.654, p < 0.01 for 5C), demonstrating that overall psychological readiness plays a central role in predicting vaccine intentions. Within the 7C model, calculation (β = 0.128, p < 0.001) and compliance (β = 0.124, p < 0.001) emerged as significant positive predictors, while conspiracy was not significantly associated with vaccination intention (β = 0.007, p = 0.819). Interestingly, the specific components' constraints, complacency, and collective responsibility did not show significant contributions when the general component was accounted for (p > 0.5), suggesting that the general readiness component largely absorbs their predictive power. In contrast, in the 5C model, calculation remained a significant predictor (β = 0.174, p < 0.001), while constraints showed a small but significant negative effect (β = −0.230, p = 0.001), and complacency had a weak positive association (β = 0.056, p = 0.046). Notably, the explained variance in vaccination intention was higher in the 7C model (R^2^ = 0.745) than in the 5C model (R^2^ = 0.515), indicating improved criterion validity for the extended 7C scale. In response to RQ, the results presented in Table 4 provide evidence supporting the criterion validity of the bifactor 7C.

**Table 4.** Criterion validity of the 7C and 5C vaccination readiness scales.

	**7C**		**5C**	
	
**Model**	**β**	**p**	**β**	**p**
**G**	0.662	< 0.001	0.654	< 0.001
**Complacency**	0.069	0.960	0.056	0.046
**Constraints**	0.520	0.566	−0.230	0.001
**Calculation**	0.128	< 0.001	0.174	< 0.001
**Collective responsibility**	0.003	0.914	0.034	0.325
**Compliance**	0.124	< 0.001		
**Conspiracy**	0.007	0.819		

**R^2^**	0.745		0.515	

Legend: G = general vaccination readiness component, β = standardized beta coefficient, p = probability value, R^2^ = coefficient of determination

## 4 DISCUSSION

The present study examined the psychometric properties of the 7C vaccination readiness scale in a Slovenian sample, providing mixed evidence for its validity and reliability. The findings reveal both strengths and notable limitations that warrant careful consideration for future applications of this instrument.

First, the construct validity of the 7C was only partially supported. The confirmatory factor analysis (CFA) of the bifactor 7C model showed somewhat weak model fit, falling just below commonly accepted thresholds. Further inspection revealed two problematic items: one from the complacency component and one from the constraints component, both of which showed inadmissible estimates, such as negative residual variances and over-saturated factor loadings. After removing these items, the model showed no further estimation issues, yet the overall model fit remained suboptimal. These results raise concerns about the structural validity and cross-sample replicability of the 7C questionnaire, as our sample yielded inadmissible estimates and sub-optimal fit, unlike previous studies (8, 4).

Second, convergent validity was assessed by correlating the latent components of the 7C model with a general conspiracy belief item. As expected, the item was strongly negatively associated with the general component, confirming that individuals with higher overall vaccination readiness are less prone to conspiratorial thinking. The conspiracy and calculation components also showed a negative correlation, while collective responsibility showed a modest positive association. These patterns provide moderate support for convergent validity, particularly for the general and conspiracy components. These findings are similar to those in the Geiger et al. (8) study, which provided support for the convergent validity of the 7C scale by finding medium-sized correlations between general vaccination readiness and its components, and COVID-19 conspiracy beliefs.

Finally, criterion validity was evaluated through regression analyses predicting vaccination intention using both 7C and 5C bifactor models. In both models, the general component emerged as the strongest predictor of vaccination intention, reinforcing the importance of general psychological readiness in vaccine uptake. In the 7C model, both calculation and compliance made significant predictive contributions. Other components—complacency, collective responsibility, and conspiracy—did not significantly predict vaccination intention once the general component was included, suggesting their effects are largely subsumed under the general readiness component. However, the 7C model explained more variance in vaccination intention than the original 5C model, supporting the incremental predictive validity of the extended scale, in line with Geiger et al. (8). The higher explained variance in the 7C model compared to the 5C model suggests that the addition of compliance and conspiracy components provides incremental predictive value beyond the original 5C framework, particularly for capturing motivational and social dimensions relevant to vaccination intention.

Taken together, our findings present a paradox: the theoretical structure of the 7C is somewhat weak, yet its practical utility remains high. This points to a need for reconceptualising certain components, such as the constraints and complacency components. This echoes concerns raised in previous validations, including the Japanese and French studies, which reported difficulties with the calculation component (4, 23). The present findings clarify the conditions under which the 7C scale can be meaningfully used. Although previous validation studies have already pointed to structural weaknesses in the scale, particularly at the level of specific components, the 7C framework remains theoretically relevant and practically useful when vaccination readiness is conceptualised as a broad, overarching psychological disposition rather than as a set of equally strong and independent subdimensions. Our results suggest that the 7C scale is most appropriately used as a measure of general vaccination readiness, supported by a dominant general factor that shows good reliability and strong predictive validity. In contrast, caution is warranted when interpreting individual component scores, especially for components such as constraints and complacency, which showed weaker and less stable psychometric properties. In such cases, the use of subscale scores may lead to overinterpretation and is therefore not recommended.

Moreover, while the general vaccination readiness component plays a dominant role in predicting vaccination intention and conspiracy beliefs, specific components such as compliance and calculation also provide meaningful evidence of convergent and criterion validity. An additional limitation of the present study concerns the mode of questionnaire administration. The 7C scale was validated using an online survey format, and it remains unclear whether the observed psychometric properties would fully generalise to alternative administration modes, such as paper-and-pencil questionnaires. Differences in response behaviour, attentional engagement, or social desirability may affect item functioning and factor structure across modes of administration.

Based on these findings, we recommend several directions for future research. First, we suggest revising the theoretical framework to be informed by the empirical factor structure, particularly by addressing the complacency and constraints components. Second, given that the study was conducted in Slovenia, our findings point to context-specific measurement limitations, rather than definitive shortcomings of the 7C framework. However, further cross-cultural validation is needed to assess whether the observed structural issues are specific to the Slovenian sample or apply more broadly. Third, qualitative research could offer deeper insight into how individuals understand and interpret vaccination decision-making processes, potentially uncovering components not captured by the current scale. Future research should also examine the measurement invariance and reliability of the 7C scale across different administration formats to ensure its robustness in diverse research and applied settings. Finally, comparative studies are needed to evaluate whether the 7C scale provides meaningful predictive advantages over the more parsimonious 5C scale, especially in applied public health contexts. Despite its structural limitations, the 7C scale shows promise as a practical tool for predicting vaccination behaviours. However, theoretical refinement is necessary to align the conceptual framework with empirical reality and enhance the scale's construct validity.

## 5 CONCLUSIONS

This study evaluated the Slovenian version of the 7C vaccination readiness scale and found mixed psychometric evidence. The scale's theoretical 7-component structure showed weaknesses, with suboptimal model fit and problematic items. These findings suggest that, although the 7C scale is a valuable tool for predicting vaccination intention, revisions to its conceptual framework, particularly regarding the complacency and constraints components, are needed to improve construct validity and cross-cultural applicability.
